# Optimizing sustainable control of *Meloidogyne javanica* in tomato plants through gamma radiation-induced mutants of *Trichoderma harzianum* and *Bacillus velezensis*

**DOI:** 10.1038/s41598-024-68365-z

**Published:** 2024-08-01

**Authors:** Mahsa Rostami, Samira Shahbazi, Reihaneh Soleimani, Abozar Ghorbani

**Affiliations:** 1 Nuclear Agriculture Research School, Nuclear Science and Technology Research Institute (NSTRI), Karaj, Iran; 2 Department of Plant Protection, College of Agriculture, Isfahan (Khorasgan) Branch, Islamic Azad University, Isfahan, 81595-158 Iran

**Keywords:** Chitosan application, Combined biocontrol approaches, Molecular identification, Nematicidal activity, Soil infection conditions, Root-knot nematodes, Microbiology, Plant sciences, Zoology, Environmental sciences

## Abstract

This study investigates the efficacy of *Trichoderma* spp. and *Bacillus* spp., as well as their gamma radiation-induced mutants, as potential biological control agents against *Meloidogyne javanica* (Mj) in tomato plants. The research encompasses in vitro assays, greenhouse trials, and molecular identification methodologies to comprehensively evaluate the biocontrol potential of these agents. In vitro assessments reveal significant nematicidal activity, with *Bacillus* spp. demonstrating notable effectiveness in inhibiting nematode egg hatching (16–45%) and inducing second-stage juvenile (J2) mortality (30–46%). Greenhouse trials further confirm the efficacy of mutant isolates, particularly when combined with chitosan, in reducing nematode-induced damage to tomato plants. The combination of mutant isolates with chitosan reduces the reproduction factor (RF) of root-knot nematodes by 94%. By optimizing soil infection conditions with nematodes and modifying the application of the effective compound, the RF of nematodes decreases by 65–76%. Molecular identification identifies *B. velezensis* and *T. harzianum* as promising candidates, exhibiting significant nematicidal activity. Overall, the study underscores the potential of combined biocontrol approaches for nematode management in agricultural settings. However, further research is essential to evaluate practical applications and long-term efficacy. These findings contribute to the development of sustainable alternatives to chemical nematicides, with potential implications for agricultural practices and crop protection strategies.

## Introduction

Root-knot nematodes (*Meloidogyne* spp.) are a type of plant parasite that cause considerable damage to crops worldwide. Among them, *Meloidogyne javanica*, Chitwood 1949^1^ (Mj) is a particularly widespread threat that can infest a wide range of host plants and cause significant yield losses. Conventional methods of nematode control, often based on chemical nematicides, pose environmental risks and raise concerns about their long-term efficacy and safety. Alternative approaches are therefore increasingly being explored, with biological control emerging as a promising avenue. Utilizing the antagonistic properties of beneficial microorganisms offers a sustainable and environmentally friendly solution to reduce nematode damage while maintaining soil health and ecosystem integrity^2^.

Microorganisms are integral to soil vitality, fostering plant growth, and development, and aiding in disease prevention. Leveraging their inherent mechanisms, these microorganisms can serve as effective biocontrol agents against various soil-borne pathogens. In addition to promoting plant growth, microorganisms can combat plant diseases by generating inhibitory compounds and triggering the plant's immune responses against pathogens. The utilization of microorganisms as biofertilizers and biopesticides represents a viable and economically appealing strategy for sustainable agriculture, with the potential for a mutually beneficial outcome^3^. Also, microorganisms can establish a symbiotic relationship with their host plants, either directly suppressing disease-causing pathogens or indirectly activating the plant's defense mechanisms for protection. Furthermore, this relationship enhances the plant's resilience to both biotic and abiotic stresses. Pathogen inhibition is achieved through a variety of mechanisms, including the production of antibiotics, lytic enzymes, phytohormones, and effective colonization^4^. *Trichoderma* spp. and *Bacillus* spp. are well-documented biocontrol agents known for their antagonistic activity against various plant pathogens, including nematodes. These microorganisms exert their effects through various mechanisms, such as competition for nutrients and space, production of antimicrobial compounds and stimulation of plant defense responses. In addition, their ability to colonize the rhizosphere and engage in mutualistic interactions with host plants further enhances their effectiveness in suppressing nematode populations^5^. Past research has illustrated the considerable efficacy of fungal spores, hyphae, and metabolites from various strains of *Trichoderma* in combating root-knot nematodes, showing significant promise^6^. Also, *Bacillus* spp. has been shown to enhance plant growth parameters and reduce root-knot nematode damage^7^.

In recent years, advances in genetic manipulation have enabled the development of mutant strains of microorganisms with improved biocontrol capabilities. Through targeted genetic modification, these mutants exhibit improved traits tailored to control specific plant pathogens, including nematodes. By exploiting this genetic diversity, the researchers aim to optimize the efficacy and sustainability of biological control strategies to combat plant parasites^8^. As a mutagenic agent, gamma radiation provides a powerful tool for generating genetic variability and new microbial variants with desired traits. By exposing mutant strains of *Trichoderma* and *Bacillus* to gamma radiation, researchers seek to improve their biocontrol effect against plant pathogens while ensuring the stability and safety of these modified organisms in agricultural ecosystems^9,10^.

This project offers a thorough investigation into the efficacy of mutant strains of *Trichoderma harzianum* and *Bacillus velezensis* Ruiz-García 2005^11^, which have been subjected to gamma radiation, for the biological control of Mj in tomato plants. It aims to propose optimal application methods to effectively mitigate root-knot nematode damage. Utilizing a combination of in vitro assays, greenhouse trials, and molecular identification methodologies, we seek to demonstrate and assess their potential as sustainable alternatives to chemical nematicides.

## Materials and methods

### Preparation of nematode and microorganisms

Roots infected with *Meloidogyne* sp. were harvested from the greenhouse in Alborz province. Using the single egg mass technique, a single egg mass was picked with a sterile needle and then placed on the tomato plant (*Lycopersicon lycopersicum* (L.) H. Karst., Deut. Fl.: 966. Nov 1882 cv. Early Urbana) root in sterile soil. After nematode reproduction on the infected plant, the eggs were subsequently extracted and incubated on tomato plant roots to ensure pure population reproduction. The extraction method for nematode eggs involved using sodium hypochlorite^12^. After rinsing with tap water, the infected roots were sliced into 2–3 cm-long segments and blended with 0.5% NaOCl solution. The blending process lasted for 30 s at low speed, after which the mixture was sieved successively through 20-, 200-, and 500-mesh/in. sieves. Eggs retained on the 500-mesh sieve were gently rinsed with water to remove NaOCl residue and collected in a Petri dish.

The perineal pattern of mature females served as the basis for identifying the root-knot nematode species. The morphological characteristics of the perineal pattern in female nematodes were examined following the identification key provided by Eisenback et al.^13^. Also, specific primers for Mj, *M. hapla*, Chitwood 1949^14^, *M. incognita*, Meng 2004^15^, and *M. arenaria*, Chitwood 1949^16^ were used for species identification. The primers used were as follows: for Mj, Fjav 5′-GGTGCGCGATTGAACTGAGC-3′ and Rjav 5′-CAGGCCCTTCAGTGGAACTATAC-3′; for *M. incognita*, Finc 5′-CTCTGCCCAATGAGCTGTCC-3′ and Rinc 5′-CTCTGCCCTCACATTAAG-3′; for *M. arenaria*, Far 5′-TCGGCGATAGAGGTAAATGAC-3′ and Rar 5′-TCGGCGATAGACACTACAAACT-3′; and for *M. hapla*, F 5′-TGACGGCGGTGAGTGCGA-3′ and R 5′-TGACGGCGGTACCTCATAG-3′^17^.

Nematode DNA was extracted from full egg masses. Egg masses were placed in 0.2 mL tubes containing 16 μL of ddH_2_O. After freezing at − 80 °C for 15 min and vortexing, 20 μL of worm lysis buffer (containing Tris–HCl, MgCl_2_, Mercaptoethanol, KCl, and Tween 20) along with proteinase K were added. Tubes were incubated at 65 °C (1 h) and 95 °C (10 min), followed by centrifugation. For PCR, species-specific primers were used. The amplification program consisted of an initial denaturation at 94 °C for 5 min, followed by 35 cycles of denaturation (30 s at 94 °C), annealing (64 °C for Mj, 54 °C for *M. incognita*, 61 °C for *M. arenaria*, and 58 °C for *M. hapla*), and extension (45 s at 72 °C), with a final extension step of 5 min at 72 °C^17^.

*Trichoderma* spp. and *Bacillus* spp., including both wild-type strains and gamma radiation-induced mutants, were obtained from the Nuclear Science and Technology Research Institute of Iran. The antagonistic ability of isolates against some plant pathogens (*Aspergillus flavus* and *Fusarium oxysporum*) has been proven^18^. Purified cultures of *Trichoderma* were cultivated on Potato Dextrose Agar (PDA) medium and preserved at 4 °C for future applications. As for the *Bacillus* strain, long-term storage was facilitated at − 70 °C in Nutrient Broth supplemented with 30% glycerol.

### Screening of microorganism’s activity against nematode

In vitro evaluation of the nematicidal ability of *Trichoderma* spp. and *Bacillus* spp.

Two isolates, *Trichoderma* NAS120 and NAS120-M44, were cultured in Potato Dextrose Broth (PDB) in 250 mL flasks for 1 week at a temperature of 28 °C ± 2 in a shaker-incubator. Subsequently, 100 mL of each culture was centrifuged for 20 min at 1500 rpm and filtered through 0.45 μm filters (Whatman™). The resulting filtered cultures were then assessed on a Potato Dextrose Agar (PDA) medium to ensure they were free from cells^19^. Filtered and cell-free cultures were used for in vitro evaluation.

To investigate the impact of bacteria (NAS-B1, NAS-B419, and NAS-B600), fungi (NAS120 and NAS120-M44), and filtered cultures of fungi on nematode egg hatching and larval mortality, 100 eggs and 100 s-stage juveniles (J2) were individually placed in one milliliter of sterile water in separate 6 cm Petri dishes. J2s were obtained from eggs that were incubated for 48 h at 28 °C in the incubator. After that time, the live J2s were collected for the test. Subsequently, 5 mL of suspension containing bacteria (10^8^ CFU/mL) and fungi (10^7^ CFU/mL), along with other treatment solutions, were added. Distilled water and Abamectin 1.8% emulsifying liquid (Aria Chemical Company) served as the control in both experiments, and the number of dead larvae was recorded after 48 h, while unhatched eggs were counted after 72 h^20^. The experiments were conducted using a completely randomized design with four replications.

### Evaluation effect of *Trichoderma* spp. and *Bacillus* spp. against nematode in greenhouse condition

#### Greenhouse test 1

Tomato seedlings were sown in 3 kg plastic pots filled with a mixture of soil (field soil and river sand, 1:2 ratio). The experiment followed a completely randomized design with four replications. When the tomato seedlings reached the four-leaf stage, they were inoculated by applying 20 mL of bacterial suspension (10^8^ CFU/mL, evaluated by spectrophotometer, Thermo Scientific, Inc., Waltham, MA, USA) and fungal suspension (10^7^ CFU/mL, counted with a hemocytometer slide, C-Slide^®^ by Curiosis Inc, South Korea ), along with other treatments, to the soil of each pot. Three days later, the roots of the plants were inoculated with 6000 eggs of Mj. Pots containing nematodes but no treatment served as controls. Additionally, to assess the treatments’ impact on plant growth, all treatments were applied without nematodes. The application of biocontrol agents was repeated every 20 days^21^. Throughout the experiment, pots were monitored daily and watered as needed. After 60 days following nematode inoculation, plant growth (Shoot dry and fresh weight) and nematode parameters were assessed. Following the harvesting of the plants, the fresh root weights were measured, which were used to calculate nematode parameters in the root system. The J2 were extracted from a mixed soil sample of 100 g from each pot using the tray method. The soil was spread evenly on paper towels in trays. These trays were immersed in shallow water and left at room temperature (25–28 °C) for 24 h. The J2s migrated from the soil through the paper towels. They were then collected using a 500 mesh sieve and counted. Additionally, the number of galls and egg masses per gram of plant roots was determined after staining with acid fuchsin. Furthermore, the eggs within 1 g of root were extracted and counted. Finally, the final population (Pf) and the reproduction factor (Rf = Pf/Pi) of Mj were calculated as the ratio of Pf to the initial population (Pi)^22^.

#### Greenhouse test 2

The experimental conditions and methodology were similar to those of the previous greenhouse test but with different treatments. In this segment, a combination of potent bacteria, effective fungus, and chitosan was employed. Chitosan solution (0.1%) was prepared using the commercial chitosan formulation obtained from Sigma Aldrich. It is low molecular weight chitosan (Sigma Aldrich) and soluble in water. Acetic acid was not used in the solution because of its negative effect on microorganisms. The chitosan solution was dissolved in water overnight using a stirrer until achieving a homogeneous mixture. Subsequently, after 24 h of incubation, fungal suspension (10^7^ CFU/mL) and potent bacterial suspension (10^8^ CFU/mL) were introduced into the solution for further utilization^23^. Additionally, a commercial biological nematocide (Tisan BT) from Royan Tisan Sam Company was included in the treatments to assess the efficacy of the effective microorganisms (0.1 mL/L).

#### Greenhouse test 3

Although the treatments were different, the conditions and methodology of this experiment were similar to those of the previous greenhouse tests. Two methods of plant infection with nematodes were implemented: (1) Cultivating plants in nematode-infested soil (field soil and river sand, 1:2 ratio), and (2) Cultivating plants in healthy soil (field soil and river sand, 1:2 ratio) and subsequently inoculating them at the four-leaf stage. To prepare pots containing contaminated soil, soil from previous cultivations was assessed and nematode counts were determined. In contaminated soil, 10 samples of 100 g each were taken after homogenizing the soil by turning it over. The number of nematodes in each sample was counted using the sieve and tray method. By averaging these counts, the amount of soil required for inoculation and production of contaminated soil was calculated. Each 3-kg pot was then filled with soil containing 6000 nematode eggs and J2s. Pots without nematodes were filled with 3 kg of healthy soil, and upon planting, at the four-leaf stage, they were inoculated with 6000 nematode eggs and larvae. Subsequently, three-leaf plants were cultivated in all pots.

**Figure 1:**
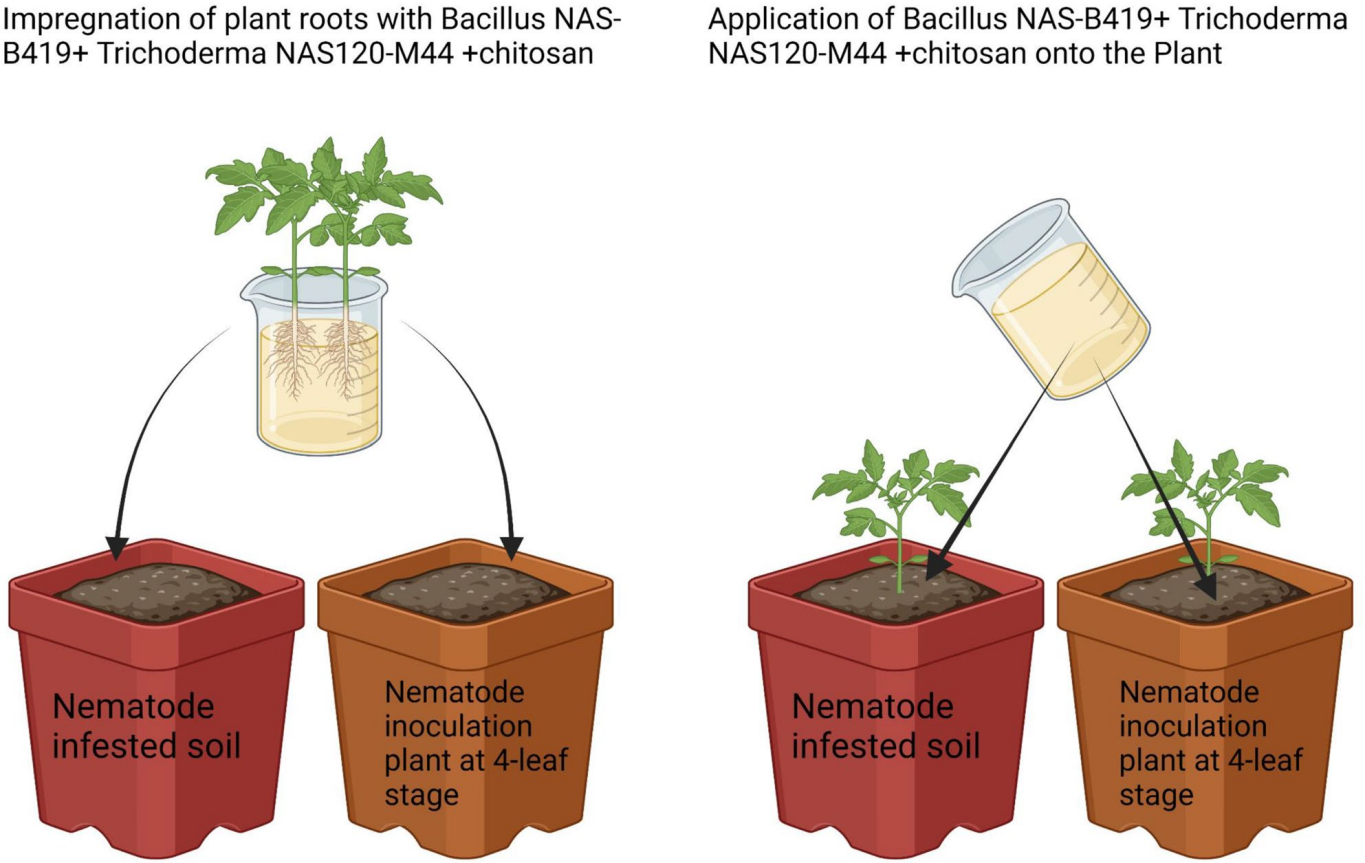
Methods of using the effective combination (*Bacillus* NAS-B419 + *Trichoderma* NAS120-M44 + chitosan) in soil infected with nematodes and inoculated soil at the 4-leaf stage of tomato plants.

For treatments involving the application of effective bacteria (10^8^ CFU/mL), fungus (10^7^ CFU/mL), and (0.1%) chitosan, where plant roots needed to be immersed in the compound, roots were submerged in 50 mL of the combined solution for 30 s before planting. Treatments requiring the mixture to be poured at the base of the plant were administered post-inoculation. These treatments were repeated every 20 days by applying the mixture at the plant base. Pots were monitored daily and watered as necessary. After 60 days post-nematode inoculation, plant growth and nematode parameters were assessed. Figure 1 visualizes these methods.

To assess the reproducibility of results, the treatments pertaining to nematode-infested soil were replicated in the subsequent greenhouse investigation. Given that plant cultivation in contaminated soil closely mirrors natural conditions, this step was crucial. The experimental setup remained identical to previous tests, and the plants were harvested 60 days post-planting and treatment application.

### Statistical analysis

The SAS statistical software (version 9.1) was employed for data analysis. Parametric indices (plant indices) were analyzed using the Proc ANOVA method, while non-parametric indices (nematode indices) were assessed using the Friedman rank test. Mean values were compared using a post hoc Tukey HSD (Honestly Significant Difference) test (P < 0.05)^24^.

### Identification of effective microorganisms

Bacterial DNA extraction was carried out using the Expin™ Combo GP DNA extraction kit from GeneAll^®^ (Tic Tech Centre, Singapore), following the manufacturer’s protocol. For fungal DNA extraction, the method described by Ghasemi et al.^25^ was employed. The quality and quantity of the extracted DNAs were assessed spectrophotometrically and adjusted to a concentration of 50 ng/μL using the Nanodrop ND-100 (Nanodrop Technologies, Waltham, Massachusetts, USA).

The universal bacterial primers 27F (5′-AGAGTTTGATCCTGGCTCAG-3′) and 1492R (5′-GGTTACCTTGTTACGACTT-3′) were used for the amplification of the complete 16S rDNA. In addition, for fungal samples, the primers 5′-CGTAGGTGAACCTGCGG-3′ and 5′-TCCTCCGCTTATTGA TATGC-3′ were used to amplify the ITS rDNA region, and the primers 5′-CATCGAGAAGTTCGAGAAGG-3′ and 5′-TACTTGAAGGAACCCTTACC-3′ were used to amplify the TEF-1α region.

For bacterial samples, the PCR reaction mixture comprised 1 μL of DNA (50 ng/μL), 1 μL each of forward and reverse primers (10 μM), 10 μL of Ampliqon^®^ Taq DNA Polymerase Master Mix Red (Ampliqon A/S, Odense, Denmark), and 7 μL of double-distilled water^26^. For fungal samples, PCR reactions were conducted in a 20 µL volume consisting of 10 µL of Master Mix, 0.2 µM of each primer for amplification of the TEF-1α and ITS regions, and 10 ng of DNA from each isolate^27^. Subsequently, PCR products were sequenced by Microsynth Company, Switzerland.

The obtained sequences were analyzed using BLASTn (NCBI: http://blast.ncbi.nlm.nih.gov). Sequences of related species and genera were retrieved from the GenBank database, and phylogenetic analysis was conducted using MEGA version 7^28^. Sequence alignment was performed using Clustal W^29^, and the Maximum Likelihood Method was employed to construct a phylogenetic tree depicting the relationships among isolates, with percentage bootstrap values derived from 1000 replicates^30^.

### Ethics approval and consent to participate

The research reported here did not involve experimentation with human participants or animals. In conducting the experimental research and greenhouse studies outlined in this manuscript, we confirm that there were no specific institutional, national, or international guidelines or legislation directly applicable to the collection and use of plant materials for our study. However, it is important to note that our research was conducted under the supervision of the Nuclear Science and Technology Research Institute (NSTRI) and the Iran National Science Foundation (INSF), ensuring adherence to ethical standards and best practices in scientific research. Additionally, it's important to mention that this project did not involve the use of mutant plants for experiments.

## Results

### Nematode identification

**Figure 2:**
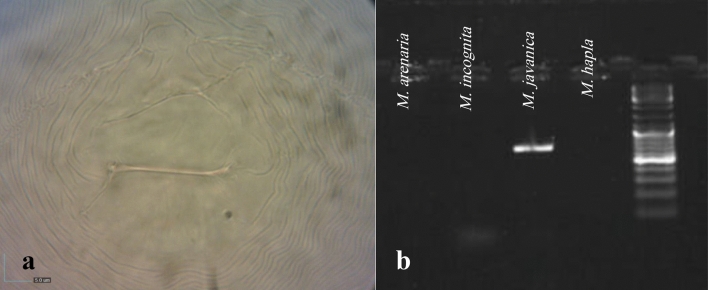
(**a**) The perineal pattern of *M. javanica*, (**b**) A 670 bp species-specific band obtained in the nematode (*M. javanica*).

The identification of the root-knot nematode species involved two complementary approaches. Firstly, the morphological characteristics of the perineal pattern in female nematodes were examined. This involved analyzing the distinct patterns and markings on the cuticle around the vulva and anus, which include the presence of a low, smooth dorsal arch and prominent lateral lines^13^ (Fig. 2a). Second, we conducted a molecular test to validate the morphological findings. Notably, a species-specific band measuring 670 base pairs (Fig. 2b) confirmed the presence of Mj, which we identified using specific primers.

### In vitro assessment of nematicidal efficacy of *Trichoderma* spp. and *Bacillus* spp.

**Figure 3:**
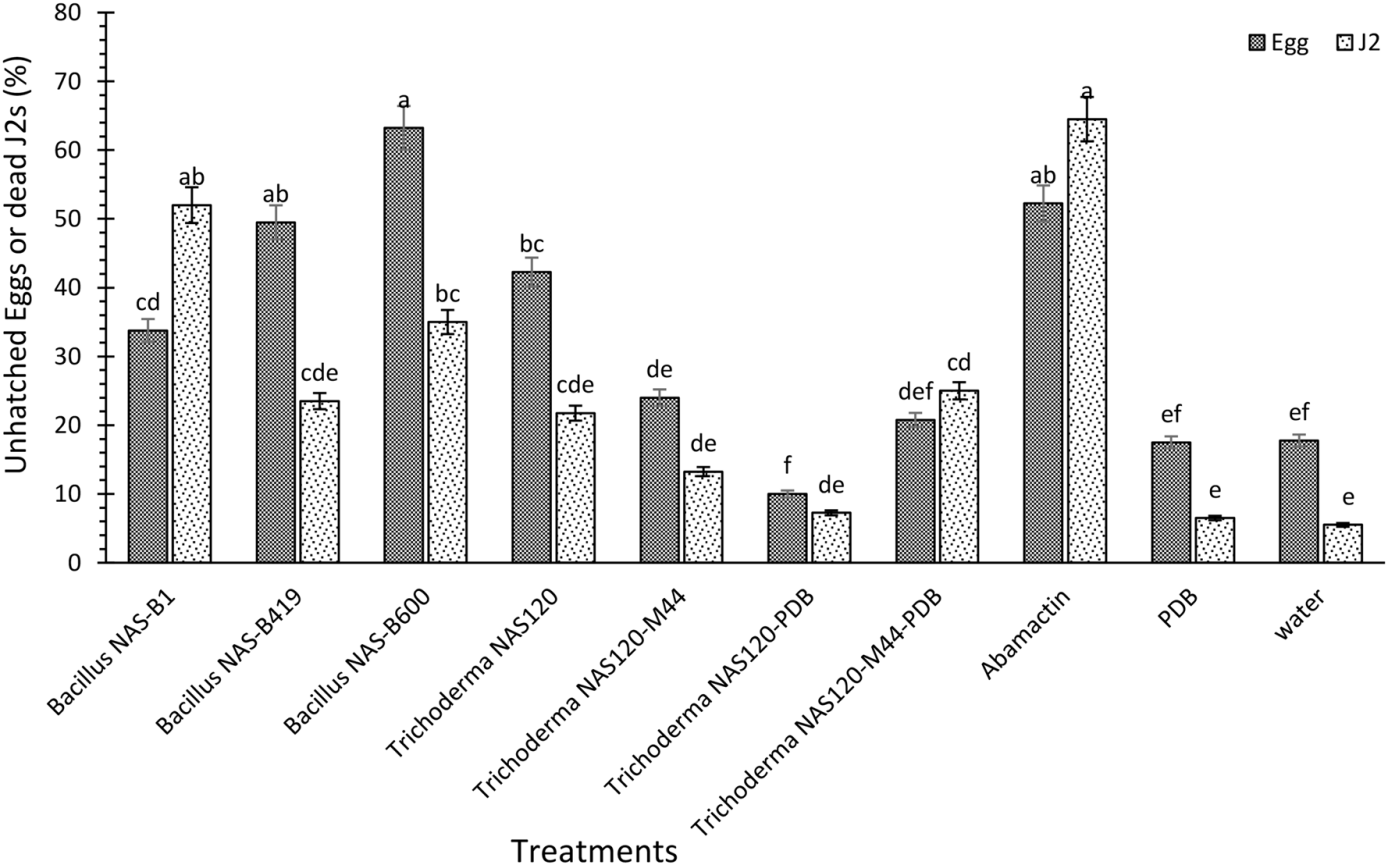
Effect of microorganisms on egg hatching and J2 mortality of *M. javanica*. Data are means of four replicates. Bars with the same letters are not significantly different (P < 0.05).

**Figure 4:**
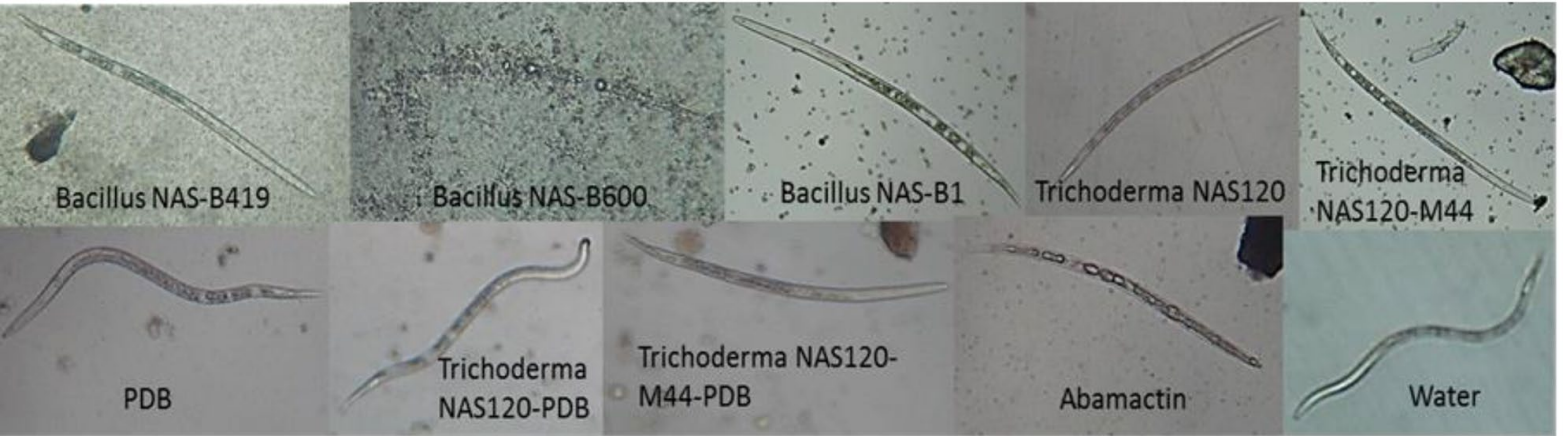
The effect of microorganisms on the J2 mortality of *M. javanica*.

The findings from the in vitro investigation revealed significant differences among treatments in terms of their efficacy in inhibiting nematode egg hatching and J2 mortality. Abamectin, *Bacillus* NAS-B1, and *Bacillus* NAS-B600 treatments exhibited notable effectiveness in both preventing egg hatching and reducing J2 mortality. Particularly, *Bacillus* NAS-B600 demonstrated the highest efficacy, reducing nematode egg hatching by 45% compared to the control. Abamectin treatment, *Bacillus* NAS-B419, *Trichoderma* NAS120, and *Bacillus* NAS-B1 also displayed considerable efficacy, reducing egg hatching by 34%, 31%, 24%, and 16%, respectively (Fig. 3). Also, Abamectin treatment, *Bacillus* NAS-B1, *Bacillus* NAS-B600, and the filtered, cell-free culture of *Trichoderma* NAS120-M44 exhibited mortality rates of 59%, 46%, 30%, and 19%, respectively, among the J2 of nematode (Fig. 3). These percentages were determined by subtracting the number of eggs or J2 in the respective treatment from those in the control. Figure 4 provides a visual representation of the J2 treated with bacteria and fungi.

### Efficacy of *Trichoderma* spp. and *Bacillus* spp. against nematode in greenhouse conditions

#### Greenhouse test 1

In the greenhouse experiments, bioagents and other treatments were applied every 20 days to align with the nematode life cycle of 45–60 days, ensuring effective disruption at various stages, and based on literature indicating optimal efficacy and resilience of microorganisms with this interval^21^.

##### Plant growth parameters

**Figure 5:**
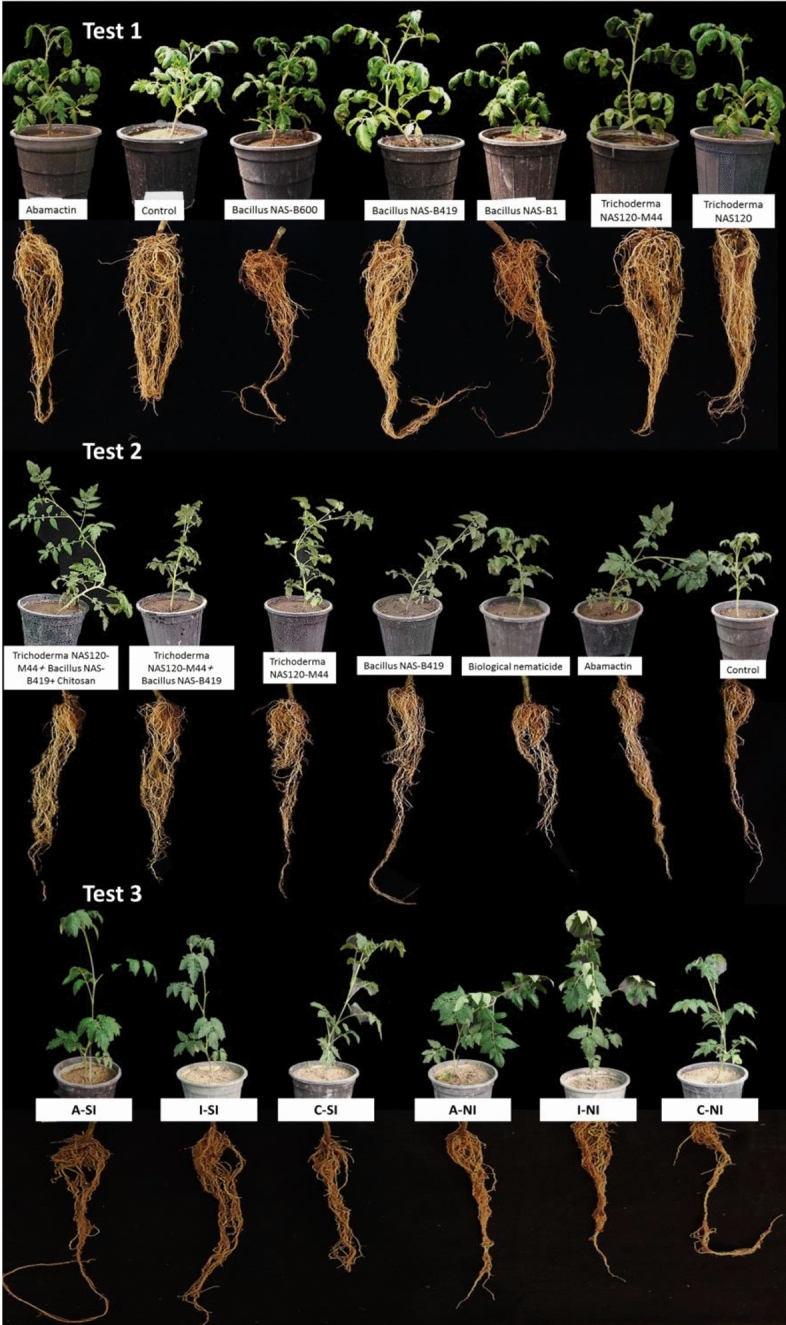
The effect of microorganisms on tomato plants infected by *M. javanica. A* Application of *Bacillus* NAS-B419 + *Trichoderma* NAS120-M44 + chitosan on the soil of the plant, *I* Impregnation of plant roots with *Bacillus* NAS-B419 + *Trichoderma* NAS120-M44 + chitosan, *C* control (water), *SI* soil infected with nematodes, *NI* nematode inoculation to a 4-leaf plant.

Among the growth indices assessed in this experiment, only the dry weight of tomato plant shoots exhibited a notable difference across treatments. Notably, treatment with mutant bacteria *Bacillus* NAS-B419 significantly enhanced the shoot dry weight of infected plants compared to the infected control plants. Conversely, the remaining treatments yielded similar effects on the shoot dry weight of infected plants, showing no significant deviation from the control (Table 1 and Fig. 5).

**Table 1:** Effects of microorganisms on tomato plant growth parameters and nematode indices of *M. javanica* under glasshouse conditions.

Treatments	Shoot dry weight (g)		Eggs/root	J2/pot soil	Galls/ root	Egg mass/root	Final population (Pf)	Reproduction factor (Rf)	Rf reduction (%)*
	With nematode	Without nematode							
*Trichoderma* NAS120	1 cd	1.62 a–d	6655 ab	750 b	329 ab	211 ab	7734 ab	1.28	73
*Trichoderma* NAS120-M44	1.92 a–d	2.52 ab	5399 b	900 b	222 bc	112 bc	6521 b	1.08	77
*Bacillus* NAS-B1	1.80 a–d	1.92 a–d	6661 ab	1950 ab	229 bc	152 abc	8840 ab	1.47	70
*Bacillus* NAS-B419	2.24 ab	2.75 a	4020 b	900 b	251 bc	120 bc	5171 b	0.86	82
*Bacillus* NAS-B600	1.67 a–d	2.10 a–c	8260 ab	1650 ab	492 ab	235 ab	10,402 ab	1.73	64
Abamectin	1.9 a–d	1.52 b–d	4741 b	750 b	127 c	80 c	5618 b	0.93	81
Water (control)	0.87 d	2.07 a–c	24,345 a	4350 a	628 a	306 a	29,323 a	4.88	–

*Reduction percent of nematode reproduction factor compared to the control.

Data are means of four replicates. Means within a column with the same letter are not significantly different (P < 0.05).

##### Nematode parameters

Abamectin, *Bacillus* NAS-B419, and *Trichoderma* NAS120-M44 treatments elicited a substantial decrease in the number of galls, egg masses, and eggs within the root system, as well as in the presence of J2 in the pot soil, ultimately leading to a reduction in the overall nematode population. Abamectin reduced the number of galls by 79%, egg masses by 73%, and the final nematode population by 81%. *Bacillus* NAS-B419 showed reductions of 60% in galls, 60% in egg masses, and 82% in the final nematode population. *Trichoderma* NAS120-M44 reduced galls by 64%, egg masses by 63%, and the final nematode population by 77%. These treatments notably reduced the nematode reproduction factor by 77% to 82%. Additionally, *Trichoderma* NAS120 and *Bacillus* NAS-B1 treatments demonstrated efficacy in diminishing the number of J2 in the soil and the occurrence of galls within the root system, respectively (Table 1). To calculate these percentages, the number for each index in the treatment group is subtracted from the number in the control group (water), then divided by the control number, and finally multiplied by 100 to obtain the percentage reduction.

#### Greenhouse test 2

##### Plant growth parameters

Analysis of both the dry and fresh weight of tomato plant shoots in this experiment did not reveal any significant differences across treatments. However, a notable distinction was observed between the group infected with nematodes and the group without nematode inoculation, reaching significance at the 5% level (Fig. 5).

##### Nematode parameters

Abamectin, *Trichoderma* NAS120-M44, and *Bacillus* NAS-B419 treatments, along with their combinations, notably reduced the number of egg masses, eggs within the root system, and J2 in the soil, leading to a significant decrease in the overall nematode population. These treatments achieved a remarkable reduction in the nematode reproduction factor, ranging from 92 to 94%. Particularly, the combination of *Bacillus* NAS-B419, *Trichoderma* NAS120-M44, and chitosan exhibited the highest reduction in the reproduction factor (Table 2). This combination reduced 77% in galls, 86% in egg masses, and 94% in the final nematode population. Additionally, Abamectin reduced galls by 76%, egg masses by 91%, and the final nematode population by 93%. In this greenhouse test, RTS-Tisan BT, a commercial biological formulation for nematode control developed by Royan Tisan Sam Company in Iran, was also used. Although it reduced the number of J2s by 97%, the number of galls by 78%, and the number of egg masses by 81%, it did not significantly reduce the Pf. In contrast, the combined fungi, bacteria, and chitosan treatment demonstrated superior efficacy in the reduction of nematode Rf, as compared to the RTS-Tisan BT.

**Table 2:** Effects of *Bacillus* NAS-B419 and *Trichoderma* NAS120-M44 and their combination with chitosan on nematode indices of *M. javanica* in the roots of tomato plants under glasshouse conditions.

Treatments	Eggs/root	J2/pot soil	Galls/ root	Egg mass/root	Final population (Pf)	Reproduction factor (Rf)	Rf reduction (%)*
*Bacillus* NAS-B419	5220 ab	4050 ab	129 ab	98 ab	9399 ab	1.56	80
*Trichoderma* NAS120-M44	2207 bc	1200 bc	77 abc	56 bcd	3484 bc	0.58	92
*Bacillus* NAS-B419 + *Trichoderma* NAS120-M44	1853 c	1350 bc	87 abc	66 bc	3290 bc	0.54	93
*Bacillus* NAS-B419 + *Trichoderma* NAS120-M44 + chitosan	1615 c	900 c	53 bc	32 de	2568 c	0.42	94
RTS-Tisan BT^2^	3656 ab	750 c	51 c	43 cde	4457 ab	0.74	90
Abamectin	2294 bc	900 c	55 bc	22 e	3249 bc	0.54	93
Water (control)	21,035 a	28,200 a	234 a	234 a	49,469 a	8.20	–

RTS-Tisan BT, Commercial Biological Nematicide, Royan Tisan Sam Company, Iran.

*Reduction percent of nematode reproduction factor compared to the control.

Data are means of four replicates. Means within a column with the same letter are not significantly different (P < 0.05).

#### Greenhouse test 3

##### Plant growth indicators

Both the dry and fresh weights of tomato plant shoots exhibited significant differences among treatments in this experiment. Specifically, treating the roots of plants with the combination of *Bacillus* NAS-B419, *Trichoderma* NAS120-M44, and chitosan resulted in a noteworthy improvement in both the fresh and dry weights of shoots in 'four-leaf stage of plant inoculated' specimens compared to 'nematode infected soil' plants. Furthermore, applying the combination to plants cultivated in infected soil also led to an increase in both the fresh and dry weights of shoots. Conversely, other treatments demonstrated similar effects on the fresh and dry weights of infected plant shoots, showing no significant deviation from the control (Table 3 and Fig. 5).

**Table 3:** Effect of the most effective combination of microorganisms (*Bacillus* NAS-B419 + *Trichoderma* NAS120-M44 + chitosan) on tomato plant growth indicators and nematode indices of *M. javanica* under glasshouse conditions.

Plant growth indicators														
Treatments	Shoot fresh weight (g)		Shoot dry weight (g)											
	NIS	NI4L	NIS	NI4L										
Impregnation of plant roots with *Bacillus* NAS-B419 + *Trichoderma* NAS120-M44 + chitosan	12.10 bc	15.62 a	2.10 abc	2.80 a										
Application of *Bacillus* NAS-B419 + *Trichoderma* NAS120-M44 + chitosan onto the Plant	14.30 ab	12.87 abc	2.25 ab	2.32 ab										
Water (control)	11.55 c	10.50 c	1.37 c	1.55 bc										

Nematode parameters														
Treatments	Eggs/root		J2/pot soil		Galls/root		Egg mass/root		Final population (Pf)		Reproduction factor (Rf)		Rf reduction (%)*	
	NIS	NI4L	NIS	NI4L	NIS	NI4L	NIS	NI4L	NIS	NI4L	NIS	NI4L	NIS	NI4L
Impregnation of plant roots with *Bacillus* NAS-B419 + *Trichoderma* NAS120-M44 + chitosan	767 bc	318 cd	1950 c	2100 c	100 abc	70 bc	27 c	35 ab	2750 c	2434 c	0.45	0.40	65	69
Application of *Bacillus* NAS-B419 + *Trichoderma* NAS120-M44 + chitosan onto the Plant	628 bc	219 d	2550 c	3300 bc	113 ab	52 c	57 ab	34 ab	3219 bc	3538 bc	0.53	0.58	59	55
Water (control)	1980 a	1100 ab	6750 a	6150 ab	142 a	155 abc	106 a	105 a	8784 a	7284 ab	1.46	1.25	-	-

Data are presented as the mean of four replicates. Means within a column with the same letter are not significantly different (P < 0.05).

*Reduction percent of nematode reproduction factor compared to the control.

*NIS* Nematode-infested soil, *NI4L* Nematode inoculation to a 4-leaf plant.

##### Nematode parameters

Impregnating plant roots with the combination of *Bacillus* NAS-B419, *Trichoderma* NAS120-M44, and chitosan in nematode-infested soil, along with inoculating plants at the four-leaf stage, resulted in a significant reduction in the final nematode population. These treatments achieved a notable decrease in the nematode reproduction factor by 65% and 69%, respectively (Table 3). Impregnation of plant roots with *Bacillus* NAS-B419 + *Trichoderma* NAS120-M44 + chitosan resulted in a 29 and 54% reduction in galls, a 66 and 74% reduction in egg masses, and a 65 and 69% reduction in the final nematode population. Applying the combination to the plant led to a 25 and 67% reduction in galls, 46 and 67% in egg masses, and 55 and 59% in the final nematode population. Given the higher rate of nematode multiplication observed in treatments related to plants grown in contaminated soil, which closely resembles natural conditions, the treatment involving the mentioned combination was repeated in contaminated soil to verify the reproducibility of the test results.

**Figure 6:**
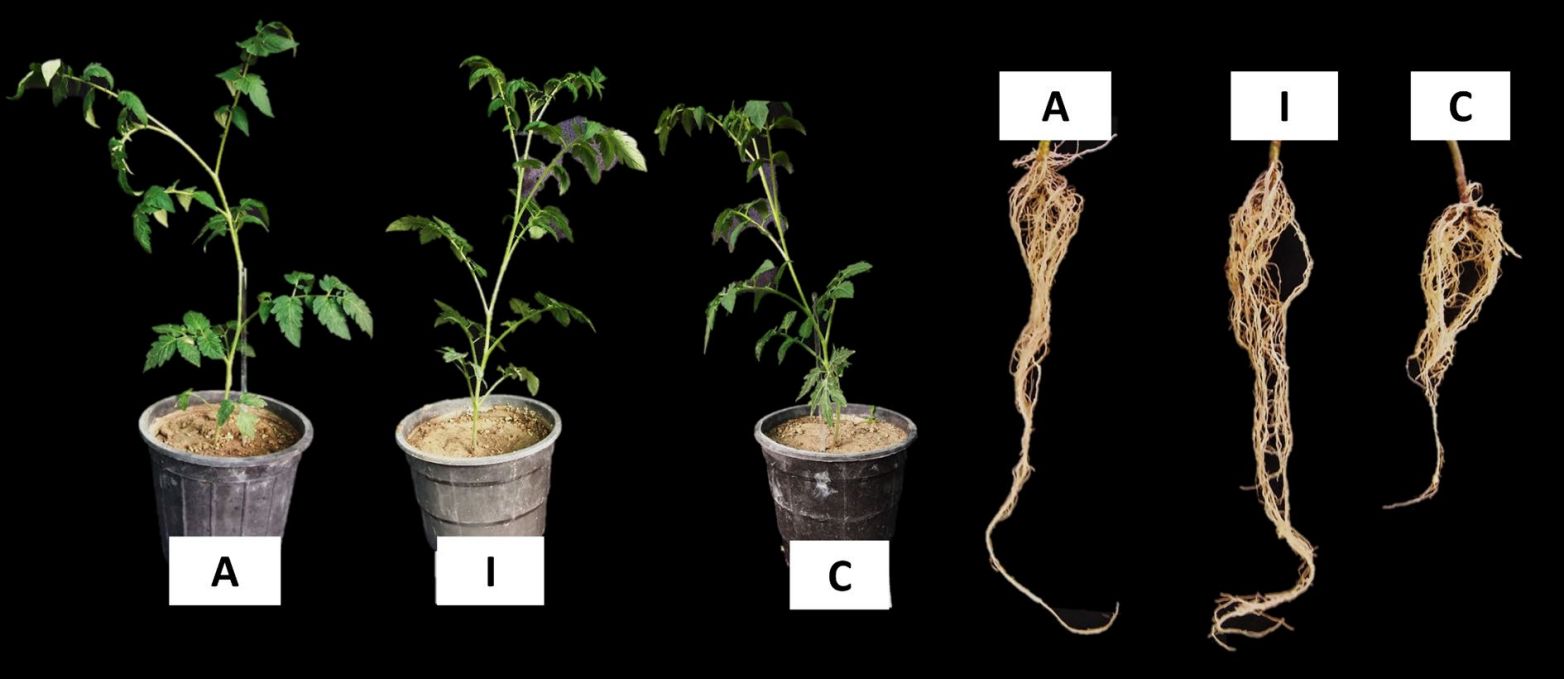
The effect of "Impregnation of plant roots with *Bacillus* NAS-B419 + *Trichoderma* NAS120-M44 + chitosan" and "Application of that combination" onto tomato plants in infected soil by *M. javanica. A* Application of *Bacillus* NAS-B419 + *Trichoderma* NAS120-M44 + chitosan on the soil of the plant, *I* impregnation of plant roots with *Bacillus* NAS-B419 + *Trichoderma* NAS120-M44 + chitosan, *C* control (water).

Repetition of the test showed that impregnation of plant roots with the effective combination of *Bacillus* NAS-B419 + *Trichoderma* NAS120-M44 + chitosan significantly in infected nematode soil improved the fresh and dry weight of plant shoots compared to infected control plants (Table 4 and Fig. 6).

**Table 4:** Effect of the most effective combination of microorganisms (*Bacillus* NAS-B419 + *Trichoderma* NAS120-M44 + chitosan) on tomato plant growth indicators and nematode indices of *M. javanica* in nematode-infected soil under glasshouse conditions.

Treatments	Shoot fresh weight (g)	Shoot dry weight (g)	Eggs/root	J2/pot soil	Gall/ root	Egg mass/root	Final population (Pf)	Reproduction factor (Rf)	Rf reduction (%)*
Impregnation of plant roots with *Bacillus* NAS-B419 + *Trichoderma* NAS120-M44 + chitosan	15.62 a	2.80 a	696 b	1350 b	78 b	29 b	2125 b	0.35	76
Application of *Bacillus* NAS-B419 + *Trichoderma* NAS120-M44 + chitosan onto the Plant	12.87 abc	2.32 ab	432 b	2100 b	79 b	33 b	2610 b	0.43	70
Water (control)	10.50 c	1.55 bc	3240 a	5550 a	167 a	97 a	8957 a	1.49	-

Data are presented as the mean of four replicates. Means within a column with the same letter are not significantly different (P < 0.05).

*Reduction percent of nematode reproduction factor compared to the control.

Both treatments, involving impregnating plant roots in nematode-infested soil with the combination of *Bacillus* NAS-B419 + *Trichoderma* NAS120-M44 + chitosan, and applying the combination directly into the soil around the plant, resulted in a decrease in the final population of nematodes. Notably, dipping the roots of plants in the solution led to a higher reduction in the reproduction factor (76%), as indicated in Table 4. Impregnating plant roots with a potent blend of *Bacillus* NAS-B419, *Trichoderma* NAS120-M44, and chitosan resulted in a remarkable 53% decrease in galls, a substantial 70% reduction in egg masses, and an impressive 76% decrease in the final nematode population. Moreover, when applied to the plant, the combination exhibited efficacy, with a 53% decrease in galls, a 66% reduction in egg masses, and a 71% decrease in the final nematode population.

### Molecular identification of effective bacteria and fungi

**Figure 7:**
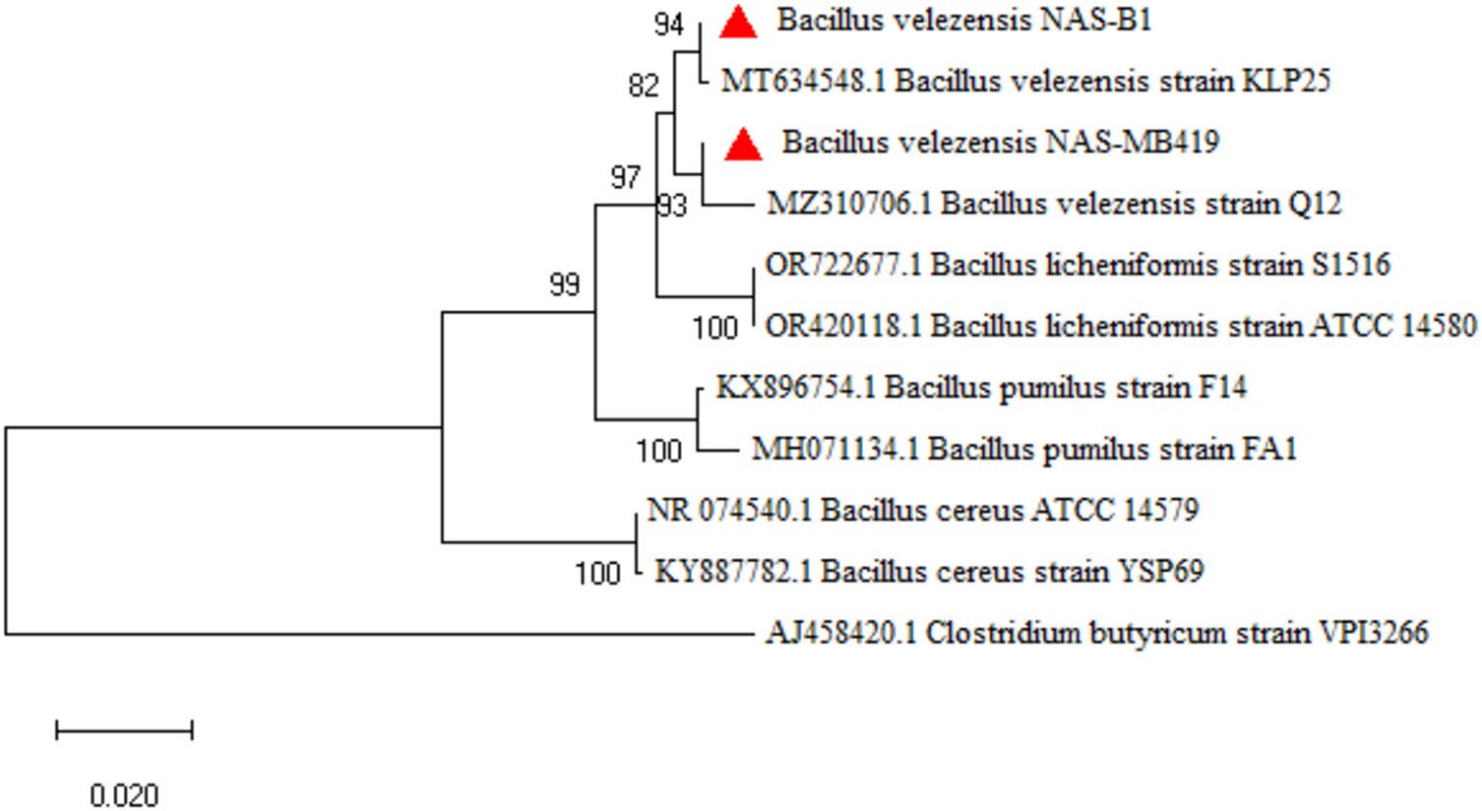
Phylogenic relationships of *Bacillus* spp. (*Bacillus velezensis* NAS-B1 and NAS-B419) based on 16S rDNA sequences (maximum likelihood method). Numbers on the branches indicate bootstrap values. *Clostridium butyricum* was used as a root.

**Figure 8:**
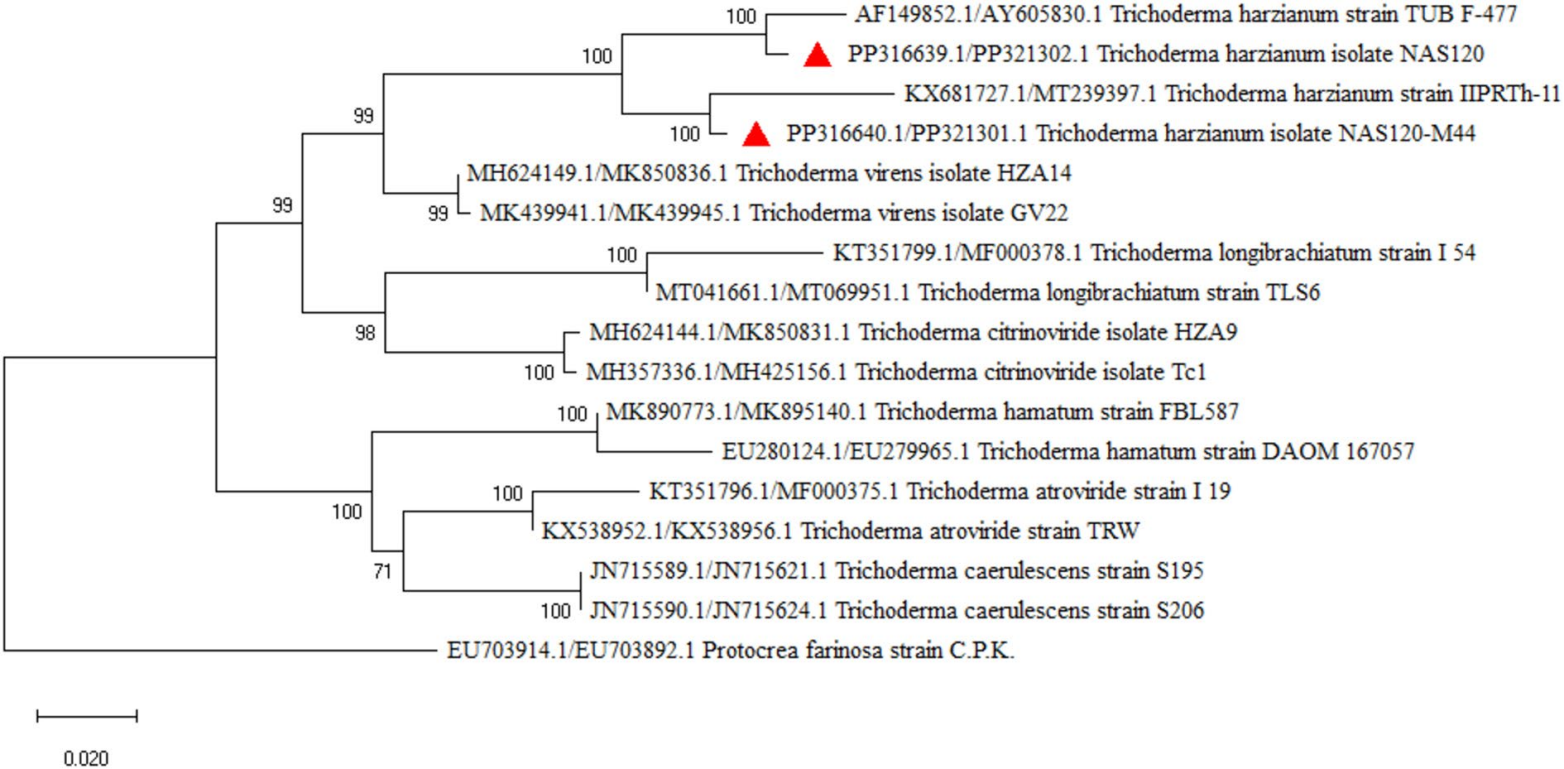
Phylogenic relationships of *Trichoderma* spp. (*Trichoderma harzianum* NAS120 and NAS120-M44) based on ITS-rDNA and TEF-1α region sequence (maximum likelihood method). Numbers on the branches indicate bootstrap values. *Protocrea farinosa* was used as a root.

Isolates were identified through a comparison of their 16S rDNA and ITS sequences with those archived in the GenBank database. The bacterial isolates were identified as *B. velezensis*, while the fungi were identified as *T. harzianum*. The sequences of their 16S rDNA, ITS-rDNA and, TEF-1α regions have been deposited in the GenBank (Table 5). The phylogenetic relationships among the identified bacterial isolates, fungi, and closely related species and genera are depicted in Figs. 7 and 8.

**Table 5:** Accession numbers of sequence data for bacteria and fungi for 16S rDNA, ITS rDNA and TEF-1α regions.

Regions sequence	Bacteria and Fungi	Accession no
16S rDNA	*Bacillus velezensis* NAS-B1	PP320414
	*Bacillus velezensis* NAS-B419	PP320416
ITS-rDNA	*Trichoderma harzianum* NAS120	PP316639
	*Trichoderma harzianum* NAS120-M44	PP316640
TEF-1α	*Trichoderma harzianum* NAS120	PP321302
	*Trichoderma harzianum* NAS120-M44	PP321301

The phylogenetic tree presented in Fig. 7 illustrates the evolutionary relationships among various *Bacillus* strains, with a particular focus on the isolates NAS-B1 and NAS-B419. These isolates are prominently marked and show a close genetic relationship with other strains within the *B. velezensis* clade. The tree is rooted with *Clostridium butyricum* as an out-group, providing context for the evolutionary divergence of the studied strains.

The phylogenetic tree depicted in Fig. 8 represents the evolutionary relationships among *Trichoderma* isolates, with a focus on ‘NAS120’ and ‘NAS120-M44’, as determined by ITS-rDNA and TEF-1α gene regions. The tree elucidates the genetic diversity within the genus, highlighting the distinct lineage of the two isolates in question (Supplementary Tables).

## Discussion

The present study aimed to evaluate the efficacy of strains of *Trichoderma* spp. and *Bacillus* spp., and their mutants that were generated through gamma radiation, for the biological control of Mj in tomato plants. Nuclear technology can be effective in the production of biological control agents and increase their useful properties. The investigation encompassed a comprehensive approach, integrating in vitro assays, greenhouse trials, and molecular identification methodologies, to elucidate the potential of these biocontrol agents as sustainable alternatives to chemical nematicides. These findings contribute valuable insights to the field of plant pathology, emphasizing the potential of *Trichoderma* and *Bacillus* mutants as sustainable nematode management tools for tomato crops. In particular, gamma-irradiated *Bacillus* NAS-B419 and *Trichoderma* NAS120-M44 showed superior efficacy in reducing nematode egg hatching, J2 mortality, and total nematode population compared to their wild-type counterparts. For example, *Bacillus* NAS-B419 reduced nematode egg hatching by 31% and achieved a mortality rate of 46% in J2 nematodes, while the wild-type strain *Bacillus* NAS-B1 showed a lower reduction in egg hatching by 16%. In greenhouse trials, the combination of *Bacillus* NAS-B419, *Trichoderma* NAS120-M44, and chitosan resulted in a remarkable 92–94% reduction in the nematode reproduction factor, significantly outperforming the wild strains. These results emphasize the potential of gamma radiation to enhance the beneficial properties of biological control agents, leading to more robust and effective biological control methods against root-knot nematodes. Introducing effective methods to combine fungi, bacteria, and chitosan in this study can help solve the problem of root-knot nematode damage in agricultural fields.

The in vitro evaluation of the nematicidal activity of *Trichoderma* spp. and *Bacillus* spp. gave promising results. Filtered cultures of *Trichoderma* NAS120-M44 showed significant antagonistic activity against J2s, while bacterial strains NAS-B1, NAS-B419 and NAS-B600 showed considerable efficacy in suppressing nematode populations. These results are in agreement with previous studies emphasizing the biocontrol potential of *Trichoderma* and *Bacillus* strains against various plant pathogens, including nematodes^19,31^. The antagonistic effect of *Bacillus* was demonstrated in an in vitro test, that significantly inhibited the hatching of nematode eggs^22^. In addition, *T. asperellum* showed considerable nematicidal activity under laboratory conditions, causing high egg-hatching suppression (96.6%) and high J2 mortality (90.3%) in *M. incognita*^19^. Previous studies have also highlighted the efficacy of *Trichoderma* spp. metabolites in reducing root-knot nematode damage^32^. In particular, the cell-free culture of *Trichoderma* NAS120-M44 resulted in J2 mortality, suggesting that the metabolites produced by this mutant strain are effective against nematodes.

Greenhouse trials provided valuable insights into the effectiveness of biological control agents under realistic conditions. They were conducted in several phases to identify the most effective combination of treatments for nematode control and plant growth enhancement. Each phase was analyzed independently to assess the efficacy of the treatments within that specific phase, ensuring that the results were reliable and not confounded by potential interactions between different experimental conditions across phases. They showed the application of mutant isolates of bacterial (B419) and fungal (NAS120-M44) suspensions significantly reduced the damage caused by nematodes to tomato plants compared to the control, resulting in a remarkable 77–92 reduction in the nematode's RF. Notably, the inclusion of treatments without nematodes allowed for the assessment of the agents' impact on plant growth parameters, revealing their potential as growth-promoting agents. Although we did not directly test for specific Plant-growth promoting rhizobacteria (PGPR) traits such as indoleacetic acid production, ammonia production or phosphate solubilization activity, our results showed a significant increase in plant weight, indicating the growth-promoting potential of the microorganisms, which is further supported by previous studies on these isolates demonstrating their extracellular enzyme production and antagonistic properties^27,33^. In subsequent greenhouse tests, the synergistic effects of a combination of strong bacteria, effective fungi and chitosan were investigated. This combined approach resulted in a remarkable 94% reduction in nematode RF, a rate comparable to that of abamectin and higher than that of RTS-Tisan BT. Sohrabi et al.^34^ reported that the combined use of *Glomus mosseae*, *B. subtilis*, and *T. harzianum* has a better effect compared to their individual use. Also, other researchers have emphasized the compatibility and biocontrol potential of *T. harzianum*, *B. subtilis*, and *P. fluorescens* Migula 1895 (Approved Lists 1980), which makes them appear as a promising tool for soilborne pathogen control^35^. In addition, chitosan, a natural biopolymer, and its derivatives have shown effective control over plant root-knot nematodes and enhancement of plant defense mechanisms against pathogens^36,37^. Furthermore, the combination of chitosan with effective bacteria has been shown to reduce root-knot nematode damage^38^. Therefore, the combination of *Bacillus* NAS-B419 + *Trichoderma* NAS120-M44 + chitosan appears to be compatible and highly effective against nematode damage.

The exploration of effective control combinations for pathogens is crucial, but equally significant is determining how to maximize their efficacy. Various methods of utilizing these microorganisms have been documented, including seed treatment, soil treatment, and seedling plant treatment^7,39,40^. Greenhouse Test 3 introduced variations in nematode inoculation methods, simulating different scenarios of nematode infestation in agricultural settings. These variations allowed for a more comprehensive evaluation of the biocontrol agents' adaptability and efficacy across diverse conditions. In this study, comparison between impregnation of plant roots with the evaluated combination and application of the combination onto the plant revealed that impregnation of plant roots was more effective in reducing the RF of nematodes. Additionally, as these methods simulate natural infection conditions more closely when applied in infected soil, the test was repeated in the infected soil. Impregnation of plant roots with the combination resulted in a 76% decrease in RF.

The molecular identification of effective microorganisms has significantly advanced the understanding of their taxonomic diversity and phylogenetic relationships. In this study, the identified fungi have been classified under the species *T. harzianum*, while the bacteria have been assigned to *B. velezensis*. These taxonomic classifications provide crucial information about the identities and genetic relatedness of the microorganisms under investigation.

*B. velezensis* is a type of gram-positive bacteria renowned for its ability to enhance plant growth. It has been documented that various strains of this species possess the capacity to inhibit the growth of microbial pathogens, spanning bacteria, fungi, and nematodes. Through genomic analysis, it has been elucidated that *B. velezensis* harbors strain-specific gene clusters responsible for the synthesis of secondary metabolites. These metabolites play pivotal roles in both suppressing pathogens and promoting plant growth. Specifically, *B. velezensis* demonstrates a robust genetic capability for producing cyclic lipopeptides (such as surfactin, bacillomycin-D, fengycin, and bacillibactin) as well as polyketides (including macrolactin, bacillaene, and difficidin). Furthermore, the secondary metabolites generated by *B. velezensis* have the potential to induce systemic resistance in plants. This mechanism enables plants to defend themselves against repeated assaults by harmful microorganisms, contributing to enhanced plant health and resilience^41^. A study has presented compelling evidence regarding the effectiveness of *B. velezensis* VB7 as both a potent nematicide and an inducer of immune responses against root-knot nematode infestation in tomato plants. Laboratory experiments demonstrated that *B. velezensis* VB7 significantly impeded the hatching of RKN eggs and notably reduced the mortality of Mj J2s by 87.95% and 96.66%, respectively. Additionally, when applied in nematode-infested conditions, *B. velezensis* VB7 triggered an immune response by inducing microbe-associated molecular pattern (MAMP)-triggered immunity, leading to the upregulation of transcription factors and defense genes. Furthermore, the study revealed the coordinated expression of various defense genes associated with immune response pathways^42^. Furthermore, six volatile organic compounds (VOCs) produced by *B. velezensis* GJ-7 demonstrated diverse modes of action against *M. hapla*, encompassing direct-contact nematicidal activity, fumigant activity, and repellent activity. As a result, these compounds show potential as promising biocontrol agents against root-knot nematodes^43^.

Numerous research findings indicate that most *Trichoderma* species can produce bioactive compounds and display antagonistic properties against plant-pathogenic nematodes. Furthermore, *Trichoderma* is employed to enhance plant growth, optimize nutrient utilization, fortify plant resistance, and mitigate pollution from agrochemicals. The mechanisms involved in the biological control of nematode diseases comprise competitive exclusion, antibiosis, antagonistic activity, and mycoparasitism, along with the promotion of plant growth and the induction of systemic resistance in symbiosis with plants^44^. The use of *T. harzianum* not only diminishes nematode populations and penetration rates but also improves plant growth, increases the content of nutritional elements and triggers systemic resistance in the plants. In addition, *T. harzianum* shows promising capabilities in the production of indoleacetic acid (IAA), exhibits remarkable ammonification activity, and shows enzymatic activities such as protease and lipase^45^. Yan et al.^46^ demonstrated that *T. harzianum* effectively suppressed *M. incognita* infestation in tomato plants, achieving a notable nematode reduction percentage of 61.88%. Their findings underscore *T. harzianum*’s beneficial role in bolstering resistance against root-knot nematodes by stimulating secondary metabolism and enhancing the activity and transcripts of defense-related enzymes in tomato plant roots. Nematode infections were observed to elevate levels of reactive oxygen species (ROS) and lipid peroxidation in tomato plant roots; however, colonization with *T. harzianum* led to a significant reduction in ROS, malondialdehyde, and electrolyte leakage. This reduction was correlated with the heightened accumulation of various secondary metabolites, including flavonoids, phenols, lignin, and cellulose.

Gamma rays serve as a means to bolster the advantageous traits of biological agents against plant pathogens. This agricultural technique has proven beneficial over the years^47,48^, contributing to the enhancement of biological agent properties^49^. The gamma mutants of *B. subtilis* UTB1, M419, and M464 have better antifungal properties against *A. flavus* than the wild type. Production of iturin-like lipopeptides and swarm motility were increased, allowing them to colonize surfaces and reduce aflatoxin to a greater extent^50^. Induced gamma irradiation also resulted in increased production of biosurfactants and biofilms in mutants of *B. subtilis* UTB1^18^. Furthermore, the enhancement of volatile production by gamma radiation in *Lactiplantibacillus plantarum*, Zheng 2020^51^ had a promising result in controlling sapstain fungi in wood stores and infected trees^52^. The antifungal metabolites of *T. harzianum*, *T. viride*, and *T. koningii* mutants were assayed by HPLC. They produced highly active exo-enzymes and had the highest isozyme band number and quantity of chitinase and beta-1,3 glucanase^53^. Moreover, the efficacy of *Trichoderma* against *Alternaria solani*, *F. oxysporium*, and *Rhizoctonia solani* was improved by the use of gamma rays, and the antagonistic activity of the second-generation variants was higher than that of the first-generation^54^. Based on our knowledge and research, this study marks the first exploration into the biological inhibitory potential of two irradiated isolates, *T. harzianum* NAS120-M44 and *B. subtilis* NAS-B419, against root-knot nematodes. In essence, these biocontrol agents demonstrate the ability to alleviate damage caused by root-knot nematodes through irradiation-induced modifications. It appears that the fungi and bacteria investigated in this study could complement each other by activating diverse resistance pathways and targeting distinct points of effect. Consequently, the concurrent application of these biocontrol agents holds significant promise for potentially substituting fertilizers and pesticides.

## Conclusion

In conclusion, this study investigated the efficacy of *Trichoderma* spp. and *Bacillus* spp., along with their gamma radiation-induced mutants, as potential biological control agents against Mj in tomato plants. The results demonstrated promising nematicidal activity of *Trichoderma* and *Bacillus* strains in vitro, leading to significant reductions in nematode populations. Greenhouse trials further confirmed the effectiveness of mutant isolates in reducing nematode-induced damage to tomato plants, especially when combined with chitosan. Molecular identification provided valuable taxonomic insights into the effective microorganisms. Specifically, *B. velezensis* and *T. harzianum* emerged as promising candidates, exhibiting significant nematicidal activity. Overall, the study underscores the potential of combined biocontrol approaches for nematode management in agricultural settings, although further research is essential to evaluate practical applications and long-term efficacy. Future research efforts may concentrate on optimizing application methods, exploring additional synergistic combinations, and assessing long-term sustainability in agricultural ecosystems. Additionally, conducting field trials under diverse environmental conditions would validate the practical utility of these biocontrol agents, facilitating their adoption as sustainable alternatives to chemical nematicides.

### Supplementary Information

Supplementary Tables.

## Data Availability

Sequence data supporting this manuscript are deposited in NCBI GenBank under accession numbers: *Bacillus* *velezensis* NAS-B1 (PP320414), *Bacillus* *velezensis* NAS-B419 (PP320416), *Trichoderma* *harzianum* NAS120 (PP316639, PP321302), and *Trichoderma* *harzianum* NAS120-M44 (PP316640, PP321301).
