# Supplementary material for: Optimizing sustainable control of Meloidogyne javanica in tomato plants through gamma radiation-induced mutants of Trichoderma harzianum and Bacillus velezensis
Source: Sci Rep. 2024 Aug 1;14:17774. doi: 10.1038/s41598-024-68365-z (PMC11294331; doi:10.1038/s41598-024-68365-z)
Supplement: Supplementary file 1 — Supplementary Tables. [file 41598_2024_68365_MOESM1_ESM.docx]

**ANOVA and Friedman Test Summary Tables for the Effects of Microorganisms on Nematode Indices under In vitro and Glasshouse Conditions**

Table S1. Effect of microorganisms on egg hatching and J2 mortality of *M. javanica*.

| **non-parametric indices** | | | | |
| --- | --- | --- | --- | --- |
| \| P-value \| \| --- \| | Test statistic | \| Error Degrees of Freedom \| \| --- \| | Source of Variation | Indices |
| <.0001 | 24.8 | 21 | treatment | Eggs |
| <.0001 | 38.01 |  |  | J2 |

Table S2- Effects of microorganisms on tomato plant growth parameters and nematode indices of Mj under glasshouse conditions.

| **Parametric indices** | | | | | | | | | | |
| --- | --- | --- | --- | --- | --- | --- | --- | --- | --- | --- |
| P-value | | F-value | | \| Mean Square (MS) \| \| --- \| | \| Degrees of Freedom (df) \| \| --- \| | | Source of Variation | | Shoot dry weight (g) |  |
| 0.0003 | | \| 4.05 \| \| --- \| | | \| 0.85052582 \| \| --- \| | 13 | | Model | |  |  |
|  | |  | | \| 0.21006429 \| \| --- \| | 42 | | \| Error \| \| --- \| | |  |  |
| **non-parametric indices** | | | | | | | | | | |
| \| P-value \| \| --- \| | Test statistic | | \| Error Degrees of Freedom \| \| --- \| | | | Source of Variation | | Indices | | |
| 0.0071 | 4.09 | | 21 | | | treatment | | Eggs/root | | |
| 0.0023 | 5.08 | |  |  |  |  |  | J2/pot soil | | |
| 0.0004 | 6.84 | |  |  |  |  |  | Galls/root | | |
| 0.0014 | 5.52 | |  |  |  |  |  | Egg mass/root | | |
| 0.0015 | 5.47 | |  |  |  |  |  | Final population (Pf) | | |

Table S3- Effects of *Bacillus* NAS-B419 and *Trichoderma* NAS120-M44 and their combination with chitosan on nematode indices of Mj in the roots of tomato plants under glasshouse conditions.

| **non-parametric indices** | | | | | |
| --- | --- | --- | --- | --- | --- |
| P-value | Test statistic | \| Error Degrees of Freedom \| \| --- \| | Source of Variation | Indices |  |
| <.0001 | 13.85 | 21 | treatment | Eggs/root |  |
| <.0001 | 9.95 |  |  | J2/pot soil |  |
| 0.0007 | 6.31 |  |  | Galls/root |  |
| <.0001 | 17.18 |  |  | Egg mass/root |  |
| <.0001 | 11.76 |  |  | Final population (Pf) |  |

Table S4. Effect of the most effective combination of microorganisms (*Bacillus* NAS-B419+ *Trichoderma* NAS120-M44 +chitosan) on tomato plant growth indicators and nematode indices of Mj under glasshouse conditions

| **Parametric indices** | | | | | | | | | | |
| --- | --- | --- | --- | --- | --- | --- | --- | --- | --- | --- |
| \| P-value \| \| --- \|  \|  \| \| --- \| | | \| F-value \| \| --- \|  \|  \| \| --- \| | | \| Mean Square (MS) \| \| --- \| | \| Degrees of Freedom (df) \| \| --- \| | | Source of Variation | | Indices |  |
| 0.0018 | | 6.10 | | 14.060 | 5 | | Model | | Shoot fresh weight (g) |  |
|  | |  | | 2.305 | 18 | | Error | |  |  |
| 0.0011 | | 6.71 | | 1.1027 | 5 | | Model | | Shoot dry weight (g) |  |
|  | |  | | 0.1644 | 18 | | Error | |  |  |
| **non-parametric indices** | | | | | | | | | | |
| \| P-value \| \| --- \| | \| Test statistic \| \| --- \| | | \| Error Degrees of Freedom \| \| --- \| | | | Source of Variation | | Indices | | |
| <.0001 | 19.02 | | 18 | | | treatment | | Eggs/root | | |
| <.0001 | 11.13 | |  |  |  |  |  | J2/pot soil | | |
| 0.0019 | 6.03 | |  |  |  |  |  | Galls/root | | |
| <.0001 | 14.79 | |  |  |  |  |  | Egg mass/root | | |
| 0.0001 | 9.75 | |  |  |  |  |  | Final population (Pf) | | |

Table S5. Effect of the most effective combination of microorganisms (*Bacillus* NAS-B419 + *Trichoderma* NAS120-M44 + chitosan) on tomato plant growth indicators and nematode indices of Mj in nematode-infected soil under glasshouse conditions.

| **Parametric indices** | | | | | | | | | |
| --- | --- | --- | --- | --- | --- | --- | --- | --- | --- |
| \| P-value \| \| --- \| | | \| F-value \| \| --- \| | | \| Mean Square (MS) \| \| --- \| | \| Degrees of Freedom (df) \| \| --- \| | | Source of Variation | | Indices |
| \| 0.0013 \| \| --- \| | | 15.18 | | 22.97 | 2 | | Model | | Shoot fresh weight (g) |
|  | |  | | 1.51 | 9 | | Error | |  |
| 0.0032 | | 11.60 | | 2.42 | 2 | | Model | | Shoot dry weight (g) |
|  | |  | | 0.21 | 9 | | Error | |  |
| **non-parametric indices** | | | | | | | | | |
| \| P-value \| \| --- \| | Test statistic | | \| Error Degrees of Freedom \| \| --- \| | | | Source of Variation | | Indices | |
| 0.0043 | 10.64 | | 9 | | | treatment | | Eggs/root | |
| 0.0009 | 10.64 | |  |  |  |  |  | J2/pot soil | |
| 0.0043 | 10.64 | |  |  |  |  |  | Galls/root | |
| 0.0043 | 14.79 | |  |  |  |  |  | Egg mass/root | |
| 0.004 | 10.64 | |  |  |  |  |  | Final population (Pf) | |
